# Health Tract, No. 158

**Published:** 1863-08

**Authors:** 


### HEALTH TRACT, No. 158.

### SAVING MINISTERS.

It has been lately proposed in the public papers, as a means of preserving clergy-men for a longer use, to a greater age, that while they are young, they should not be expected to do so much, as is now required of them; that for the first five years of their ministry, only one sermon on the Sabbath should be given. Not one minister in a million is ever disabled by hard study, or dies prematurely from that cause. A far better plan would be to require them to preach every day and Sunday too, for the first years of their ministry, and "as ye go, preach;" take circuits, and preach in destitute places, five, or ten, or fifteen miles apart; a sermon a day on an average, the year round; and two or three on Sundays, the oftener the easier; the advantages are, that they would become acquainted with the country; would be brought into personal contact with a great variety of persons; would see human nature in its multitudinous phases; and thus in after-life would be able to read a book, more instructive to them than any other, except the Bible; and reading it well, would put in their hands a key which would unlock the human heart, and give them so complete an access to it, that the people would say: "Never man spake like this man." "He told me all that ever I did." Patrick Henry owed his greatest power to what he learned of human nature by talking to all sorts of people in his little country store. Another advantage is, that this daily active out-door life, breathing the pure air for almost all of daylight, would enable them to work off that diseased bodily condition, which is generated in theological seminaries; and would so knit and compact the constitution, so renovate it, not only by the exercise, but by the change of food and association, as to lay the foundation for many years of healthfulness in the future. It is impossible for an intelligent man to doubt for an instant, that four or five years spent, in riding every day on horseback, in the open air, with the accompanying and exhilarating mental exercise required in preaching, would be as certain to build up the constitution, as spending from morning until night in confined rooms, and eating heartily all the time, without any systematic exercise, would pull it down, and destroy it. There is nothing perplexing, or mystic, or mind-racking in ordinary ministerial duty; it is more of calm contemplation, like that of the natural philosopher, the longest-lived of all other classes, as statistics say; they study the works of God; the clergy study his word; which is a surer "word of prophecy" and a plainer. The destroyers of our clergy are not hard study; not the difficulties connected with their calling; but reckless and unnecessary exposures; irregular efforts; wrong habits of eating; unwise neglect of wholesome bodily exercises; bad hours of study, and a criminal inattention to the securement of those bodily regularities, which are indispensable to health the world over. Preaching often, does not kill; look at the Whitefields and the Wesleys and multitudes of others like them; confinement even, does not kill: Baxter and Bunyan and many more lived in jails for years together, and that too without opportunities of exercise—for their living was plain, and that not over-abundant, nor tempting either!

